# Flexibility and resilience of great tit (*Parus major*) gut microbiomes to changing diets

**DOI:** 10.1186/s42523-021-00076-6

**Published:** 2021-02-18

**Authors:** Kasun H. Bodawatta, Inga Freiberga, Katerina Puzejova, Katerina Sam, Michael Poulsen, Knud A. Jønsson

**Affiliations:** 1 Natural History Museum of Denmark, University of Copenhagen, Copenhagen, Denmark; 2 Biology Centre of Czech Academy of Sciences, Institute of Entomology, Ceske Budejovice, Czech Republic; 3 Faculty of Science, University of South Bohemia, Ceske Budejovice, Czech Republic; 4 Section for Ecology and Evolution, Department of Biology, University of Copenhagen, Copenhagen, Denmark

**Keywords:** Bacterial communities, Community flexibility, Community resilience, Gut symbionts, Illumina MiSeq, 16S rRNA gene

## Abstract

**Background:**

Gut microbial communities play important roles in nutrient management and can change in response to host diets. The extent of this flexibility and the concomitant resilience is largely unknown in wild animals. To untangle the dynamics of avian-gut microbiome symbiosis associated with diet changes, we exposed *Parus major* (Great tits) fed with a standard diet (seeds and mealworms) to either a mixed (seeds, mealworms and fruits), a seed, or a mealworm diet for 4 weeks, and examined the flexibility of gut microbiomes to these compositionally different diets. To assess microbiome resilience (recovery potential), all individuals were subsequently reversed to a standard diet for another 4 weeks. Cloacal microbiomes were collected weekly and characterised through sequencing the v4 region of the 16S rRNA gene using Illumina MiSeq.

**Results:**

Initial microbiomes changed significantly with the diet manipulation, but the communities did not differ significantly between the three diet groups (mixed, seed and mealworm), despite multiple diet-specific changes in certain bacterial genera. Reverting birds to the standard diet led only to a partial recovery in gut community compositions. The majority of the bacterial taxa that increased significantly during diet manipulation decreased in relative abundance after reversion to the standard diet; however, bacterial taxa that decreased during the manipulation rarely increased after diet reversal

**Conclusions:**

The gut microbial response and partial resilience to dietary changes support that gut bacterial communities of *P. major* play a role in accommodating dietary changes experienced by wild avian hosts. This may be a contributing factor to the relaxed association between microbiome composition and the bird phylogeny. Our findings further imply that interpretations of wild bird gut microbiome analyses from single-time point sampling, especially for omnivorous species or species with seasonally changing diets, should be done with caution. The partial community recovery implies that ecologically relevant diet changes (e.g., seasonality and migration) open up gut niches that may be filled by previously abundant microbes or replaced by different symbiont lineages, which has important implications for the integrity and specificity of long-term avian-symbiont associations.

**Supplementary Information:**

The online version contains supplementary material available at 10.1186/s42523-021-00076-6.

## Background

The establishment and maintenance of symbiosis between hosts and their gut microbiota are pivotal in the evolutionary history of animals, where microbial symbionts play a multitude of roles in nutrient management, host development and host immunity [1–3]. Diet and host taxonomy are major drivers of assemblies of gut microbial communities across diverse animal taxa, including insects [4–7], spiders [8], fish [9, 10], frogs [11], mammals [12, 13], and birds [14–18]. In vertebrates, most work to understand the importance of these factors stems from mammals and birds [12, 17, 19, 20], with intriguing differences between the two [19, 20]. Although microbiome compositions in mammals are affected by diet [21–26], mammalian gut microbiomes are tightly associated with host phylogeny [19, 20], often accompanied by taxon-specific diets [12]. In contrast, although bird taxonomy, such as host family, is associated with certain microbiome characteristics [15, 16], microbial compositions tend to not strongly associate with host phylogeny [16, 20]. In line with this, bird microbiomes exhibit higher individual variation, conceivably caused by dietary, environmental and social factors [14, 27–31]. It has been proposed that gut adaptations associated with flight (e.g., a smaller gut and consequently shorter retention time of food in the intestines) may explain the lack of strong phylogenetic signal in avian microbial community compositions [20], which are potentially intensified by compressions or expansions of dietary niches associated with latitudinal and altitudinal migrations [32–34], seasonality [35, 36], and breeding vs. non-breeding seasons [37–39].

A flexible gut microbiome that accommodates changing diets may be important for the evolutionary success of birds, but our knowledge of associations with diet changes remains sparse. For example, although gut microbiomes of migratory birds differ between wintering, stopover and breeding sites, likely due to changes in diet availability [30, 40–44], the impact of diet has not been explicitly investigated in these studies. Intra-specific differences in bird populations from habitats with potentially different food availabilities further support the plastic nature of bird gut microbiomes [28, 31, 45–50], as does the higher individual variation and gut bacterial diversity of omnivorous birds compared to bird species with more specialized diets (e.g., insectivores) [14]. The gap in pinpointing the impact of diet on gut microbiomes has to some extent been filled by recent diet manipulation studies of *Passer domesticus* (house sparrows) [51] and *Parus major* (great tits) [52], in which gut microbiomes respond according to dietary contents. If dietary changes are regular (e.g., seasonality and migration) we would expect gut microbiomes to cyclically change over time depending on temporal changes in food availability [24, 53]. The resilient nature (i.e., the recovery potential after dietary fluctuations) of gut microbiomes has been documented in several mammals [22, 24, 26, 53], but has not been explored in birds.

**Fig. 1:**
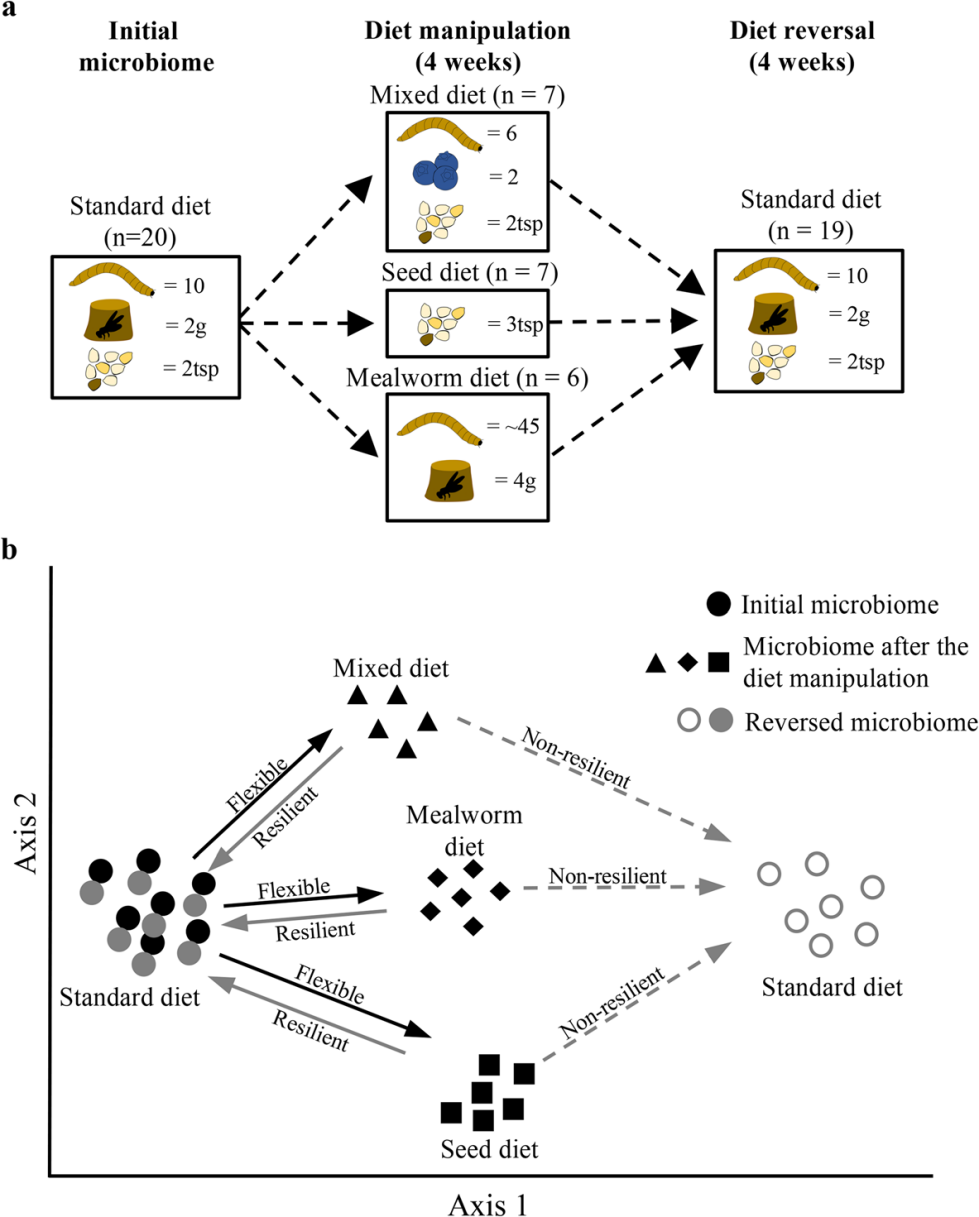
**a**. Contents of standard, mixed, seed and mealworm diets, with a schematic timeline of the experiment. **b**. Predicted flexible (toward three diet groups) and resilient (recovery after the diet reversal) microbiome responses to dietary changes in *Parus major*. Filled grey circles represent recovery of gut microbiomes if the communities are resilient, while open grey circles represent potential outcomes if microbiomes are not resilient

Here we examine the recovery potential of gut microbiomes of *P. major* after diet manipulation through characterizing gut community composition using MiSeq amplicon sequencing of the 16S rRNA gene. After feeding birds a standard diet, we fed them either a mixed (including fruits, seeds and mealworms), a seed, or a mealworm diet for 4 weeks (Fig. 1a). Subsequently, we reversed the diet-manipulated birds to the standard diet for 4 weeks to test the recovery potential of their gut microbial communities, and to assess whether full or partial microbial community restoration was achieved (Fig. 1a). If gut communities are flexible and respond to dietary contents, we expect to find both marked differences between the initial gut microbiomes and the gut microbiomes following diet manipulations, and between the three diet groups (Fig. 1b). We further hypothesised that if the gut communities are resilient to dietary changes, gut microbiomes should return to their initial community structure after the reversal period (Fig. 1b). In contrast, if the communities are not resilient, we would expect compositions to differ from initial communities after the diet reversal (Fig. 1b).

## Results

After analysing and quality filtering of sequences with DADA2 [54] within Qiime2 [55], we acquired a total of 2,965,765 16S rRNA gene sequences (mean ± SE: 20,313 ± 3407) from 169 cloacal swabs from all 9 weeks (initial first week, 4 weeks of diet manipulation and 4 weeks of diet reversal). Sequences were identified using the SILVA 132 database [56] and assigned to 2537 amplicon sequence variants (ASVs) with 100% similarity (Additional file 1: Table S1). The majority of the subsequent analyses was only conducted on data from week 1 (initial microbiomes), week 5 (microbiomes after the diet manipulation), week 9 (reversed microbiomes), since our main interest was to investigate the end result of the diet manipulation and the diet reversal. Samples from week 1 (18 samples after removing samples that failed during sequencing), week 5 (mixed diet: six samples, seed diet: five samples and mealworm diet: five samples) and week 9 (12 samples) had 1,709,192 bacterial 16S rRNA gene sequences (mean ± SE: 37,156.35 ± 5478.41) belonging to 1290 ASVs (Additional file 2: Table S2). Overall, *Firmicutes* dominated the cloacal microbiomes with 75% of the ASVs, followed by *Proteobacteria* (10.13%), *Tenericutes* (10.79%) and *Bacteroidetes* (1.83%). Only 0.64% of the sequences were unidentified at the phylum level. The initial gut microbiomes of captively raised and wild caught adults fed with identical diets (see Methods) did not differ significantly (permutational multivariate analysis of variance (PERMANOVA)_10,000 permutations_: F_1,16_ = 0.9828, *R*^*2*^ = 0.0579, *p* = 0.4175). Furthermore, the sex of the birds did not have a significant impact on the initial microbial community composition (PERMANOVA_10,000 permutations_: F_1,16_ = 1.119, *R*^*2*^ = 0.0653, *p* = 0.2806); thus, we pooled individuals irrespective of their sex in subsequent analyses. Due to the large variation in the number of sequences per sample (min: 1045 sequences and max: 224,835 sequences), we rarefied the original data set (Additional file 2: Table S2) to have 1045 sequences per sample (Additional file 3: Table S3) and performed alfa and beta diversity analysis on both the complete and rarefied data sets.

### ASV richness and diversity decrease due to diet manipulation

**Fig. 2:**
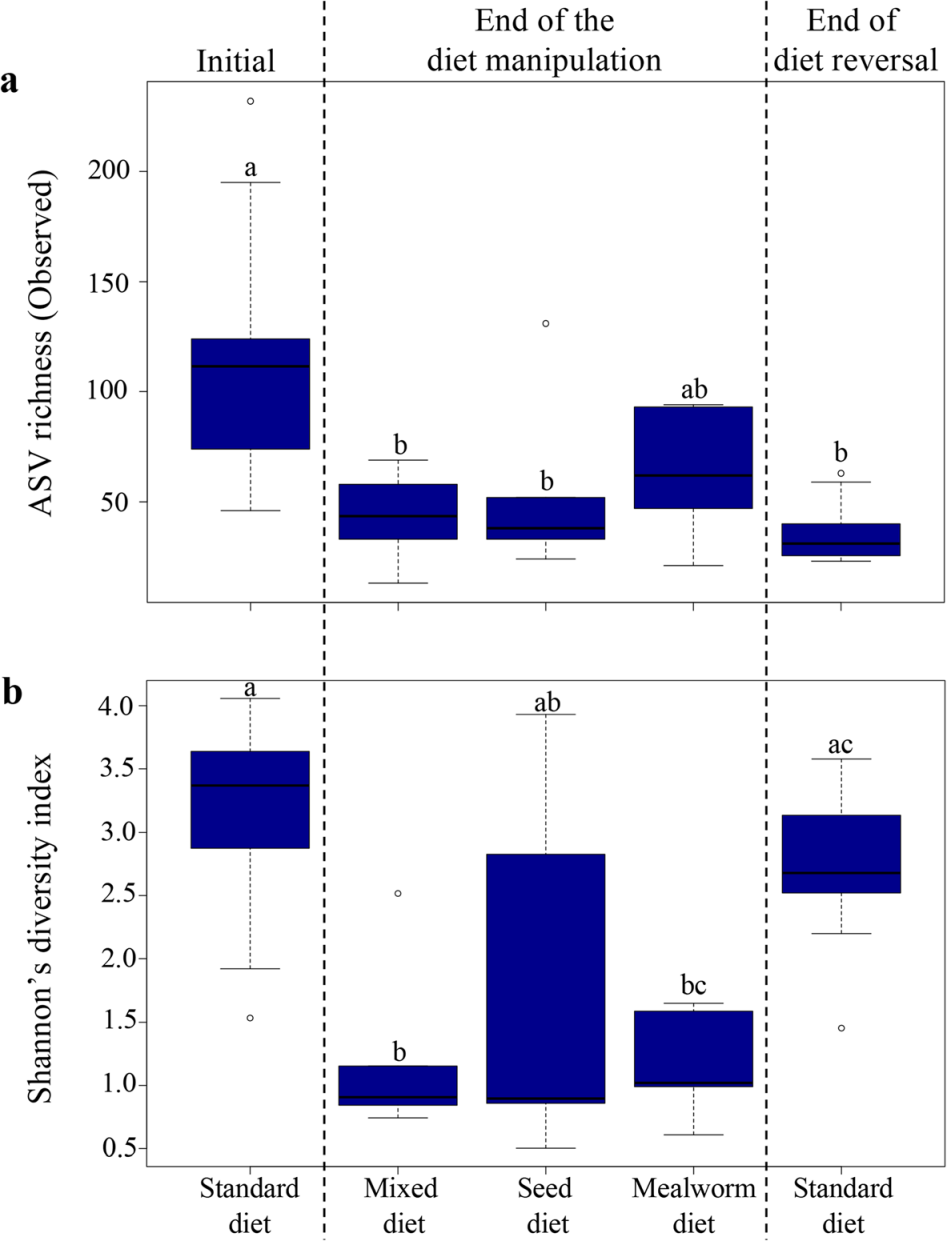
Mean ASV richness (**a**) and Shannon’s diversity index (**b**) of gut microbial communities in initial, after the diet manipulation and after the diet reversal. Letters above each boxplot represent the pairwise differences between different groups (Dunn’s post-hoc test) and different letters indicate significant differences

Bacterial richness (total number of ASVs) was significantly lower in all diet groups and in the reversed diet gut communities compared to the initial communities (Kruskal-Wallis: H = 24.89, df = 4, *p* < 0.0001; Fig. 2a, Additional file 4: Table S4). The Shannon’s diversity index was also significantly lower in all diet groups compared to the initial gut communities, but did not differ between the initial and the reversed diet communities (Kruskal-Wallis: H = 22.375, df = 4, *p* < 0.0001; Fig. 2b, Additional file 4: Table S4). We found no significant difference in richness and diversity between the three diet groups at the end of the diet manipulation period (Fig. 2, Additional file 4: Table S4). Only 27 ASVs were shared among all diet groups and many ASVs were lost or reduced in abundnace below detection after the diet manipulation and reversal (Additional files 5 and 6: Figures S1, S2). The rarefied data set provided similar results for bacterial richness (Kruskal-Wallis: H = 27.49, df = 4, *p* < 0.0001; Additional file 7: Figure S3a, Additional file 4: Table S5) and Shannon’s diversity index (Kruskal-Wallis: H = 22.19, *p* < 0.0001; Additional file 7: Figure S3b, Additional file 4: Table S5).

### Bacterial communies change with diet content

**Fig. 3:**
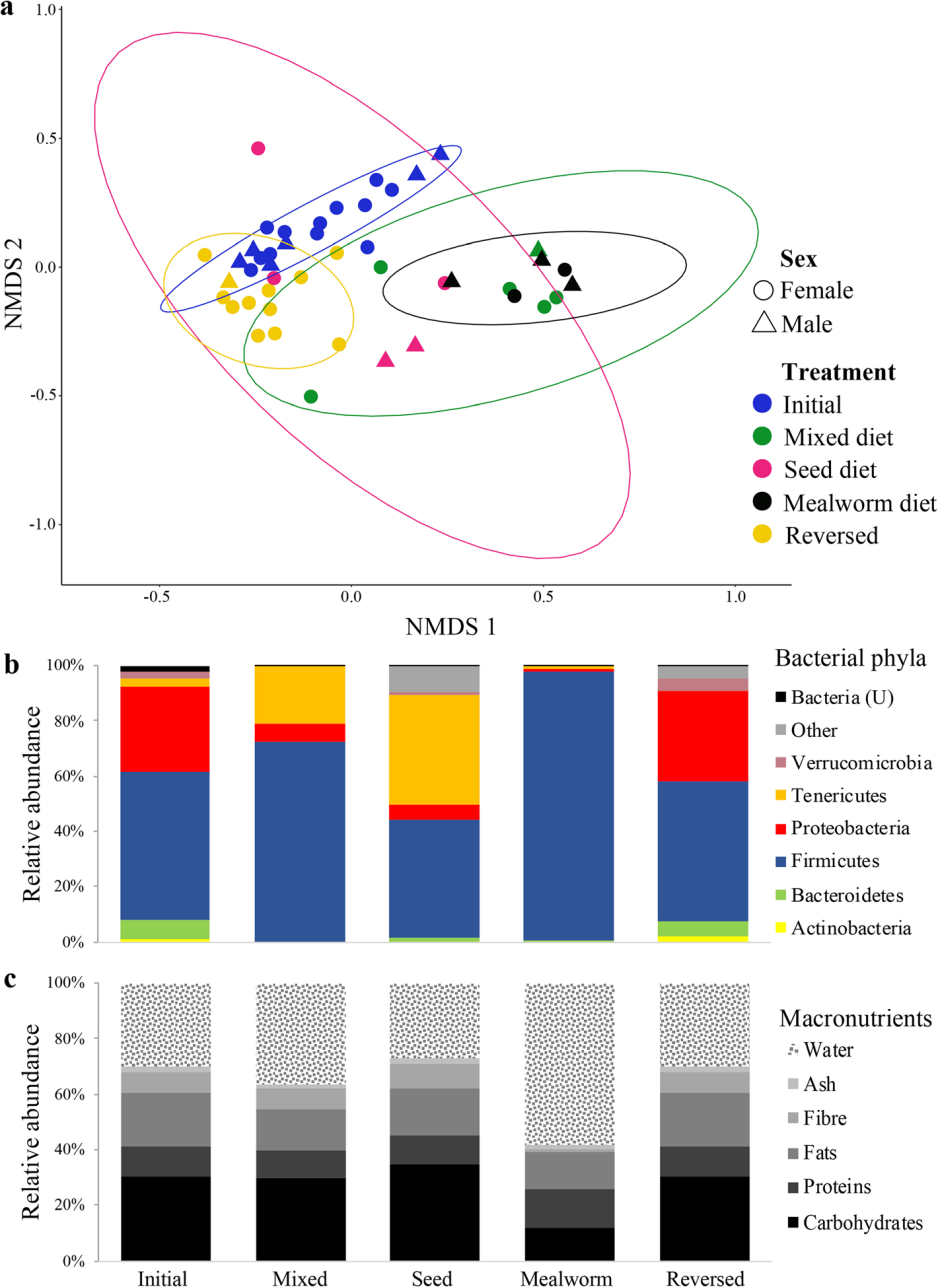
**a.** Non-Metric Multidimensional Scaling (NMDS) plot of bacterial communities for initial, mixed, mealworm, seed, and reversed diets (ellipses indicate 95% CI; stress = 0.166). Shapes represent the sex of each individual. **b.** Relative abundances of bacterial phyla in gut microbiomes under different diets. **c.** Relative abundance of macronutrients in 100 g of each diet

**Fig. 4:**
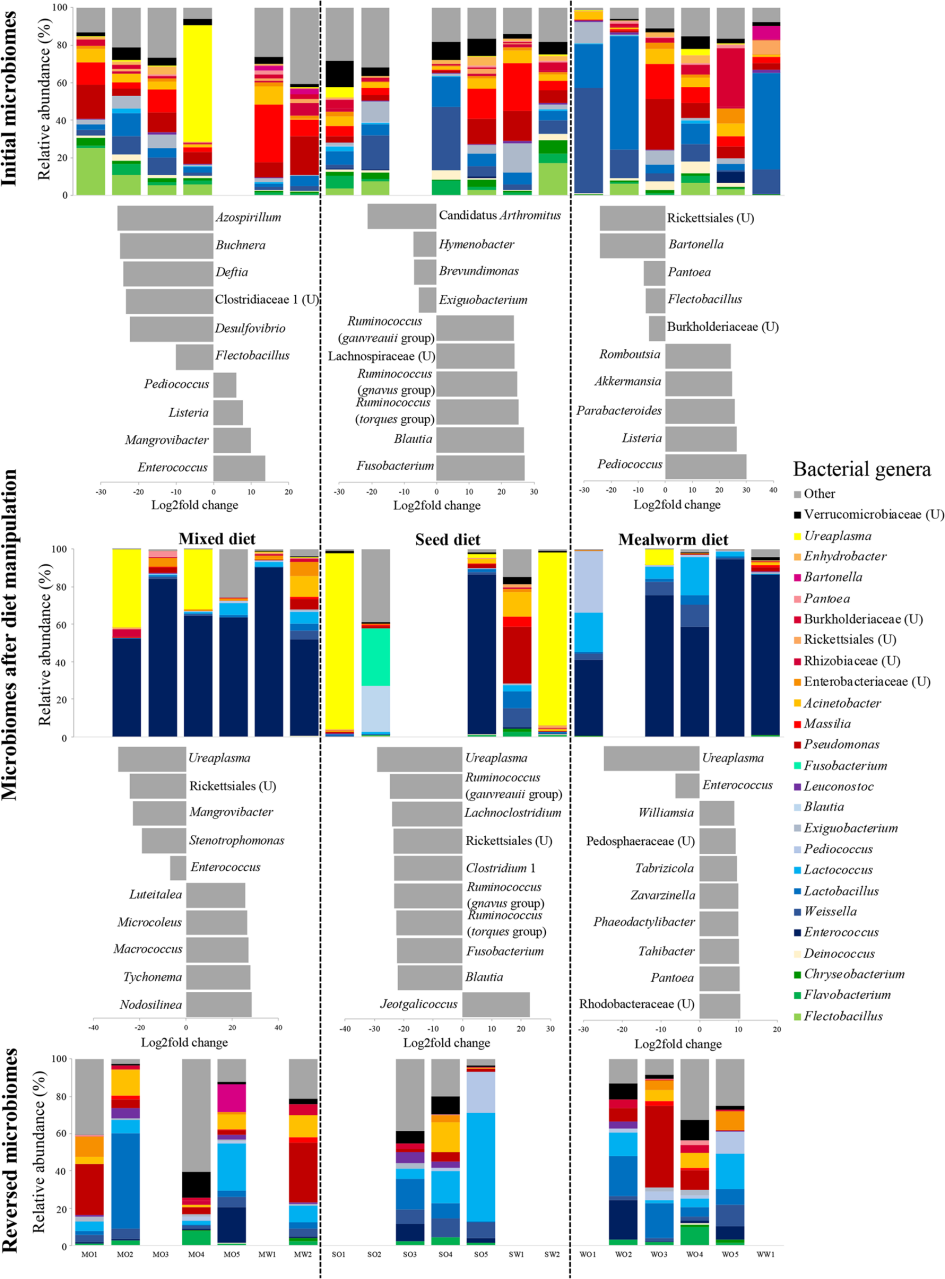
Relative abundances of the 25 most common bacterial genera in the cloacal swabs of *P. major* on the initial diet, after diet manipulation and after diet reversal are shown in the bar graphs. Unclassified genera are indicated with a “U”, following the closest taxonomic level classification (i.e., family or order). Individual birds are represented with their name codes on the x-axis. Top ten significantly differentially abundant genera (divided between comparison groups) are given between panels. Positive log_2_-fold values represent increase of certain genera in bottom panel compared to the top panel. Empty columns indicate samples that did not sequence or failed the quality filtering step

A PERMANOVA analysis demonstrated a significant impact of diet on the gut bacterial community structure, accounting for 27.1% of the observed variance (PERMANOVA_10,000 permutations_: F_4,41_ = 3.805, *R*^*2*^ = 0.271, *p* < 0.001; Fig. 3a). Pairwise PERMANOVAs showed significant deviation of bacterial communities of birds on the mixed and the mealworm diets from the initial community structure (Table 1). Gut communities of birds on the seed diet were more variable between individuals and did not differ significantly from the initial diet (Fig. 3a, Table 1). The bacterial community structure did not differ significantly between the three diet groups (Table 1). There was a significant difference between the microbiome composition of the initial and the reversed communities (Table 1, Fig. 3a), despite similarities in relative abundances of bacterial phyla and major genera between these two communities (Figs. 3b and 4). We observed significant differences in individual variation (average distance to group centroid) in microbial communities in birds on different diets (Permutation test for multivariate dispersions_10,000 permutations_: F = 4.857, df = 4, *p* = 0.0049). Pairwise comparisons revealed that gut communities of individuals on the mealworm diet exhibited significantly lower average distances to the group centroid compared to individuals on the initial, the seed and the reversed diet (Additional file 8: Figure S4), indicating that the mealworm diet reduces individual variation in gut microbial communities.

**Table 1:** Results of pair-wise PERMANOVAs with 10,000 permutations comparisons of gut bacterial communities between the initial diet, the three diet manipulation treatments, and the reversed diet (on original data set). Significant differences are indicated with asterisk (*) signs

Diet pairs	F statistic	R^**2**^	Adjusted ***p*** value
Initial vs. mixed diet	4.894	0.1819	0.0009*
Initial vs. mealworm diet	6.261	0.2297	0.0019*
Initial vs. seed diet	2.127	0.0921	0.1369
Mixed vs. mealworm diet	1.001	0.1001	1.000
Mixed vs. seed diet	1.929	0.1765	0.3579
Mealworm vs. seed diet	3.015	0.2737	0.0819
Reversed vs. mixed diet	4.465	0.2182	0.0009*
Reversed vs. mealworm diet	6.258	0.2944	0.0019*
Reversed vs. seed diet	2.181	0.1269	0.0189*
Initial vs. reversed diet	3.768	0.1186	0.0009*

Community-level analyses on the rarefied data set produced similar results, demonstrating the importance of diet on microbial community structure (PERMANOVA_10,000 permutations_: F_4,41_ = 5.439, *R*^*2*^ = 0.3467, *p* = 0.001; Additional file 7: Figure S3c). For the complete data set (including all 9 weeks), we observed a week-by-week change in gut communities, with the biggest difference occurring after 4 weeks (Additional file 9: Figure S5). The microbial communities changed more gradually over the diet manipulation (Additional file 9: Figure S5 a, c, e) and diet reversal (Additional file 9: Figure S5 b, d, f) periods.

The dominant bacterial phyla changed notably to different diets (Fig. 3b) compared to gut communities of birds on the initial diet. Initial communities were dominated by *Firmicutes* (53.45%), followed by *Proteobacteria* (30.62%), *Bacteroidetes* (6.94%) and *Tenericutes* (3.41%). In all three diet treatments, we found a decrease in *Proteobacteria*. In the mixed and the seed diet we found an increase of *Tenericutes* (Fig. 3b). In the mealworm diet treatment, *Firmicutes* vastly dominated the gut microbiota (97.5%) (Fig. 3b). However, the gut communities recovered to initial proportions of bacterial phyla after the diet reversal (Fig. 3b).

The macronutrient content of the four diets (standard, mixed, seed, and mealworm) were not markedly different, except for the mealworm diet (Fig. 3c). The standard and mixed diets were similar in their macronutrient content, except for decreased amounts of proteins and fat in the latter. The seed diet had higher fat and fibre proportions than the mixed and mealworm diet (Fig. 3c), while the mealworm diet had higher proportion of protein and less carbohydrates than the other three diets (Fig. 3c).

### Differential abundances of bacteria varied according to diet treatment

Relative abundances of the top 25 bacterial genera differed between the initial diet, the three diet manipulation treatments, and the reversed diet (Fig. 4). These 25 genera also accounted for 96.1% of all sequences. We found more variation between individual birds in the initial and the reversed bacterial communities than in the mixed and the mealworm diet treatments, whereas the high variation between communities in the seed diet remained (Figs. 4 and S4).

Significantly differentially abundant bacterial genera (using the original data set) between diet treatments were identified using DESeq2 [57]. Bacterial taxa that significantly increased during the diet manipulation also differed between the three diets (Fig. 4, Additional file 10: Table S6). Individuals on the mixed diet experienced significant increases in relative abundance of four genera (e.g., *Enterococcus*, *Mangrovibacter*, *Listeria* and *Pediococcus*) and significant decreases in 20 genera compared to their initial gut microbial communities (Additional file 10: Table S6.1). Diet reversal of mixed diet individuals led to a significant increase in 49 genera and a significant reduction in five genera, including *Enterococcus* and *Mangrovibacter* (Additional file 10: Table S6.2). The seed diet led to significant increases in 13 bacterial genera (e.g., *Fusobacterium*, *Blautia* and *Ruminococcus*), and four genera significantly decreased from initial communities (Additional file 10: Table S6.3). Diet reversal of seed diet fed individuals led to significant decreases in 20 genera and an increase in one genus (*Jeotgalicoccus*) (Additional file 10: Table S6.4). Fifteen bacterial genera increased in individuals on the mealworm diet, while five genera decreased significantly (Additional file 10: Table S6.5). After the diet reversal of individuals on the mealworm diet two genera decreased significantly (*Ureaplasma* and *Enterococcus*) while eight genera increased (Additional file 10: Table S6.6). Intriguingly, only 13% (± 6.5 SE) of the same bacterial genera that decreased during the diet manipulation increased significantly after the diet reversal (Fig. 4). However, around half of the bacterial genera (52.2% ± 23.1 SE) that increased significantly due to the diet manipulation decreased after the diet reversal (Fig. 4, Additional file 10: Table S6).

## Discussion

Exploration of host-gut microbiome dynamics of wild hosts is important to better understand the flexibility, resilience and long-term associations of symbiotic interactions under dietary changes. By examining the flexibility and resilience of wild omnivorous passerine bird gut microbiomes through diet manipulation, our findings document a rapid and significant impact of diet, aligning with other studies on passerine birds [51, 52]. We observed a significant deviation in microbial community structure from the initial gut microbiomes after diet manipulation, but we found no significant differences in gut communities between the three diet groups (Fig. 3). Despite this, there were noteworthy diet-associated trends in the bacterial community variation and differentially abundant bacterial genera. Furthermore, gut microbiomes partially recovered after the diet reversal, implying that the association between *P. major* and their gut communities is somewhat resilient to diet-induced changes.

### *P. major* gut microbiomes respond to diet changes, but not as flexibly as predicted

The deviation of gut microbial communities from the initial diet to three different diet groups exemplifies the ability of *P. major* to respond to dietary changes. However, the magnitude of the microbial response varied among diets, suggesting that responses occur according to both the physical (ratios of different diet components) and the macronutrient compositions. Although the macronutrient content of standard, mixed and seed diets did not differ markedly, differences in gut microbial communities indicate that the physical content of diets can impact the gut microbiomes (Figs. 3 and 4). A diet that is both compositionally and macronutritionally different, such as the mealworm diet (Figs. 1a and 3c), has the strongest and most consistent impact on microbiome structure (Figs. 3a and S4).

The differences in individual variation in gut microbiomes between seed and mealworm diets provided some intriguing insights into gut microbiome dynamics (Additional file 8: Figure S4). Microbiomes of birds fed with mealworms experienced significantly lower individual variation than birds fed with a standard or a seed diet (Additional file 8: Figure S4). The reduced individual variation in insectivores has been demonstrated in wild birds [14] and in another diet manipulation study of *P. major* [52], suggesting that a strictly insectivorous diet (mealworms) might be associated with a specific and narrower set of gut microbes [58] compared to the seed diet. This is potentially associated with more homogeneous nutrient availability in the mealworm diet [58] compared to the more heterogeneity from multiple types of seeds (e.g., sunflower seeds [59], millet [60], and wheat [61]). Additionally, the taxonomic diversity of initial gut microbiomes of *P. major* differed markedly from the other diet manipulation study [52], where *Proteobacteria* dominated the gut microbiomes, indicating regional or population differences. However, the observed similarity in the overall community-level response of gut microbiomes for individuals on seed or insect diets in the two studies suggest that despite these differences, microbial communities respond in similar ways to similar diet changes. The microbes responding to these diet shifts may depend on the taxonomic composition of the starting microbiome. This species-level regional variation in gut microbiomes and the flexible nature of passerine microbiomes to dietary changes further support the lack of [16, 20] or weak [62] association between bird gut microbiome structure and host phylogeny.

We also documented significant diet-specific responses of a few bacterial genera in different diet groups (Figs. 1, 3 and Additional file 10: Table S6). The seed diet led to a significant increase in relative abundance of the genera *Fusobacterium* (*Fusobacteria*), *Blautia* (*Firmicutes*), and *Ruminococcus* (*Firmicutes*). Some *Fusobacterium* members are animal pathogens [63], but their consistent presence in wild bird guts [2, 14, 64, 65], especially in herbivorous species [64–66], suggests a possible beneficial role. Bacteria from the genus *Blautia* may facilitate the metabolism of plant secondary metabolites [67, 68], consistent with an increase in the relative abundance of this genus in birds feeding on seeds. *Ruminococcus* bacteria (e.g., the *R. gauvrauii* and *R. gnavus* groups) that increased significantly with seed diet are in the family *Lachnospiraceae* and their possible functions are poorly resolved [69]. However, the *Ruminococcus gnavus* group has been proposed to degrade mucins [69], and the mucin levels in digestive tracts tend to increase on plant-based diets with high fibre contents [70]. These bacteria may thus be opportunistic and utilize the increased amount of mucins.

Birds on the mealworm diet experienced a significant increase in the relative abundance of e.g., *Rombutisia* and *Akkermansia* that are generally presumed to be associated with protein metabolism [71]. Relative increases in the *Firmicutes* orders *Lactobacillales*, *Bacillales* and *Clostridiales* (Additional file 10: Table S6.5) in birds on this diet are consistent with a previous study on insectivorous passerines [14]. Most of these lineages are believed to play roles related to protein fermentation and degradation of toxic by-products from protein metabolism [68, 72–74], thus enabling hosts to sustain an insect diet. Furthermore, a study has demonstrated prebiotic effects of mealworms on mice gut microbiomes, where mealworm exuviae led to an increase in lactic acid bacteria (e.g., family *Lactobacillaceae*) [75], similar to what we observe in *P. major* (Figs. 1a, 3c and 4). However, understanding how macronutrient content affects wild bird gut microbiome lineages and their associated functional roles requires further studies with nutritionally more distinct diets to decipher their association with gut microbial processes.

Although we cannot rule out that foodborne microbes could impact gut microbial community structures, as has been shown for a small fraction of lactic-acid bacteria from fermented foods in humans [26], we find this unlikely to be a main driver of our results. We did observe new bacterial ASVs in the three diet groups and reversed microbiomes compared to initial communities (Additional files 5 and 6: Figures S1 and S2), suggesting that new lineages could colonise bird gut microbiomes along with dietary changes. However, the most abundant ASVs observed in the manipulated gut microbiomes were shared with the initial communities (Additional file 1: Table S1 and Additional file 6: Figure S2), supporting that major changes in gut microbiomes are accounted for by pre-existing community members. Previous studies on wild birds have demonstrated low bacterial diversity and community composition differences in the midgut region (stomach and small intestine) compared to the crop microbiota. This suggests that the highly-acidic conditions in the midgut region acts as a barrier for environmental and foodborne bacteria [14, 66, 76, 77]. This is further evident from previously published mealworm gut microbiomes [78], where only a small fraction of the microbiome consists of bacterial genera that we identified in the gut microbiomes of mealworm-fed *P. major*. Furthermore, cloacal swabs appear to adequately capture the microbial diversity of entire digestive tracts [79], suggesting that the observed differences in the cloacal microbial communities are unlikely to be driven by foodborne microbes.

### Wild-bird gut microbiomes are partially resilient to diet-induced changes

For the first time, we illustrate the resilience of wild bird gut microbiomes to diet changes. However, the recovered bacterial communities did not completely mirror the initial gut communities, despite the recuperated bacterial diversity. There were differences in relative abundances of dominant bacterial genera compared to initial community compositions, and ASV richness was significantly lower in reversed than initial gut communities (Figs. 2, 4 and Additional file 5: Figure S1). Intriguingly, we found a consistent pattern that the majority of the taxa that significantly increased in abundance during specific diet treatments decreased again when birds were returned to the standard diet, while taxa that decreased significantly during the diet manipulation rarely recovered following diet reversal (Additional file 10: Table S6, Fig. 4). However, the partial recovery of gut microbiomes may also be an artifact of the short duration (4 weeks) of the diet reversal in our study. In the wild, exposure to particular diets reflecting seasonality is conceivably longer. Thus, future studies with extended diet reversal times, ideally mimicking known seasonal changes, may better reflect the effect on gut microbiomes of natural dietary changes.

Partial recovery suggests that ecologically relevant diet changes render available gut niches that are subsequently filled by either the original microbes or are replaced by novel symbiont lineages. Lineages filling particular niches may have the opportunity to flourish by outcompeting bacteria playing similar roles due to functional redundancies inherent in complex gut communities [68, 80, 81]. To understand whether this indeed plays a role, exploration of microbial gene expression between initial and reversed gut bacterial communities is needed. Our results also demonstrate that *P. major* microbiomes are resilient to natural dietary changes that individuals might experience due to seasonality (e.g., a seed-dominated diet during winter and an insect-dominated diet in spring) [36], but these recovery trajectories can be impacted by the competition among bacterial lineages that fill the same functional niches. A partial restoration of gut bacterial communities after dietary changes imply that natural diet changes could lead to symbiont losses (Additional file 5: Figure S1), further suggesting that the nature of digestive tract microbiotas in wild birds is to some extent plastic. Hosts may utilize other mechanisms, such as acquiring bacteria from the environment [40] or hosting bacteria with similar metabolic capacities [68, 80] to maintain stability after disruptions of their symbiotic associations. The flexible yet resilient nature of avian gut microbiomes may thus provide an additional level of plasticity for bird hosts to cope with natural (i.e., seasonality, migration, and interspecific competition) [32–39] and anthropogenic (i.e., habitat degradation and invasive species) [82, 83] dietary fluctuations, as predicted by many avian gut microbiome studies [28, 30, 40–49].

## Conclusions

Our study documents responses of bird gut microbiomes to diet changes and the resilient nature of gut bacterial communities, supporting the important role of gut bacterial communities in accommodating the dietary breadth of wild bird hosts. The extent of these changes implies an extra level of plasticity in hosts to adjust their dietary niche. This malleable nature of bird gut microbiomes may be causal to the observed relaxed association between bird gut microbial communities and host phylogenies. Furthermore, changes that bird gut microbiomes experience due to dietary fluctuations raise concerns about drawing conclusions on wild bird gut microbiomes based on single time points, as they imply that seasonal and regional fluctuations in food sources most likely affect gut microbial compositions that would go unnoticed in the absence of sampling across time and space. The partial recovery of gut microbiomes also suggests that niches that are opened due to diet changes are subsequently either filled by the microbes originally filling these niches or by other symbiont lineages with similar functions or metabolic properties. This suggests that symbiont loss and replacement may be prominent in bird gut microbiomes, with implications for the integrity, specificity, and long-term dynamics of avian-gut microbial associations.

## Methods

### Bird collection and captive raring

Fifteen *P. major* chicks were collected from nests at the age of 10 days in the surrounding of Ceske Budejovice, Czech Republic on 20th of May 2018 and hand raised in captivity in a breeding room at the Faculty of Sciences, University of South Bohemia. Individuals were fed with a standard diet including 10 mealworms, 2 g insect cake (a bread-like diet made from: Nutribird a21, commercial chicken food (Country’s Best Show 1 crumble), eggs, wheat flower, sugar and sunflower margarine, mixed and baked for ~ 40 min. at ~ 180 °C) and 2tsp of moistened mixed seeds (Living World Premium Mix for Cockatiels & Lovebirds) per day. Furthermore, vitamin water (Acidomid exot®) was provided three times per week. During June and July, the birds were used in a behavioural test (30 min per day), during which they were tested for behavioural responses to plant volatile compounds. The behavioural tests did not affect food intake of the birds and their feeding regime. Five additional adults were captured 20th of September 2018 and kept in captivity for 5 days on the same standard diet until the diet manipulation started. All birds were kept in individual cages (0.7 × 0.4 × 0.5 m) that were cleaned daily.

The sex of the birds was determined through extracting DNA from 10 μl of blood using the Genomic DNA Mini Kit (Geneaid Biotech Ltd., New Taipei, Taiwan) and conducting PCR using avian-specific sex primers P2 and P8 [84] following an established protocol [85]. Heterogametic females (two bands after PCR) and homogametic males (one band) were identified through visual inspection of agarose gels.

### Feeding experiment

Birds (15 captively reared and five wild-caught) were randomly divided into three compositionally different diet groups on the 26th of September 2018 and each group was offered either a mixed diet (six mealworms + two berries +2tsp mixed seeds), a mealworm diet (6 g mealworms [40–50] + 4 g insect cake) or a seed diet (3tsp mixed seeds) (Table 2, Fig. 1a). Before the introduction of different diets, a cloacal swab (using a Copan mini swab™) was collected from each individual and stored in 60 μl RNAlater® to investigate the composition of gut microbiota prior to diet manipulation. Birds were under the three different diets for 4 weeks and cloacal swabs were collected each week. At the end of the fourth week, all birds were reverted to the standard diet for an additional 4 weeks to investigate whether microbial communities recovered to their initial composition (Fig. 1a). Cloacal swabs were again collected weekly. All the swab samples in RNAlater® were stored at − 20 °C until the DNA extractions.

**Table 2:** Individual IDs of *P. major* and their corresponding rearing conditions, diet treatments and sex

Individual ID	Rearing conditions^**a**^	Diet treatment	Sex
MO1	Captive reared	Mixed diet	F
MO2	Captive reared	Mixed diet	F
MO3	Captive reared	Mixed diet	M
MO4	Captive reared	Mixed diet	F
MO5	Captive reared	Mixed diet	F
MW1	Wild caught	Mixed diet	F
MW2	Wild caught	Mixed diet	F
SO1	Captive reared	Seed diet	M
SO2	Captive reared	Seed diet	F
SO3	Captive reared	Seed diet	F
SO4	Captive reared	Seed diet	F
SO5	Captive reared	Seed diet	F
SW1	Wild caught	Seed diet	F
SW2	Wild caught	Seed diet	M
WO1	Captive reared	Mealworm diet	M
WO2	Captive reared	Mealworm diet	F
WO3	Captive reared	Mealworm diet	F
WO4	Captive reared	Mealworm diet	M
WO5	Captive reared	Mealworm diet	F
WW1	Wild caught	Mealworm diet	M

^**a**^Captive reared: individuals were taken from wild nests and reared in the breeding room; wild caught: individuals were captured as adults a week before the diet manipulation experiment was initiated

### Nutrient contents of different diets

Macronutrient contents of 100 g of different diets were analysed at the Eurofins Steins Laboratory (Vejen, Denmark). Amount of crude proteins, fats and fibre were calculated using multiple chemistry protocols at the Steins Laboratory. The amount of Nitrogen-free extract (a proxy for total Carbohydrates) were calculated through subtracting proteins, fats, fibre, ash (i.e., minerals) and water from 100 g. Ash (which include molecules that are not associated with macronutrients) was measured by burning the whole sample under 550 °C.

### Molecular methods

DNA from all the swabs were extracted using the Qiagen DNeasy Blood and Tissue® kits (Qiagen, Germany), to sequence the bacterial communities using the V4 region of the 16S rRNA gene. Manufacture’s extraction protocol was followed exactly, except for an extended incubation period (~ 14 h) during the lysis step and use of 70 μl of heated (56 °C) AE buffer during the elution step. Initial PCRs were conducted using two primers targeting the V4 region of the 16S rRNA: ‘SB711 (5’-CAAGCAGAAGACGGCATACGAGATTCAGCGTTAGTCAGTCAGCCGGACTACHVGGGTWTCTAAT-3 ‘) and ‘SA504 (5′-AATGATACGGCGAC CACCGAGATCTACACCTGCGTGTTATGGTAATTGTGTGCCAGCMGCCGCGGTAA-3 ‘) [cf. 14] and following a well establish PCR protocol for the primer pair [79]. PCR products were visualized on a 2% agarose gel. DNA from positively amplified samples from the initial PCRs along with three negative controls were sent to the Microbial System Molecular Biology lab at the University of Michigan, where the samples were sequenced (using same primers) on the Illumina MiSeq platform.

### Data analysis

Out of the 176 samples, 169 (96%) were successfully sequenced and the negative controls did not amplify or sequence, demonstrating no contaminations during DNA extractions. Sequences were analysed using DADA2 pipeline [54] within QIIME2 [55]. Bacterial 16S rRNA gene sequence were assigned to taxonomy using SILVA 132 bacterial reference library [56], and archaeal, mitochondrial and chloroplast sequences were removed subsequently. Sequences with 100% similarity were categorized into the same Amplicon sequence variants (ASVs). ASVs with fewer than 10 sequences and samples with fewer than 1000 sequences were removed from the data analysis. Further analyses were mainly conducted on samples from week one (initial gut microbiota), week five (gut microbiota after diet manipulation) and week nine (gut microbiota after diet reversal), since we were primarily interested in the final responses of gut microbiota to specific diets and their ability to recover after the diet reversal (Additional file 2: Table S2). Samples from other weeks were only analysed to investigate the trajectories of changes in bacterial communities and data is presented in Supplementary Table 1.

The data was analysed using R 3.5.3 [86, 87]. Shannon diversity index (accounting both bacterial richness and abundances) for samples were calculated using the package vegan [88]. Non-metric multidimensional scaling (NMDS) plots, permutational multivariate analysis of variance (PERMANOVA with 10,000 permutations) and pair-wise PERMANOVA comparisons were conducted using vegan [88] and the wrapper package pairwiseAdonis [89] using Bray Curtis distances between communities to identify statistical differences in gut bacterial communities and to visualize community level differences. We further investigated the individual variation in gut microbial communities through calculating the distance of each community to the centroid of their diet group, using the betadisper function in the package vegan in R [88]. Groups with a smaller average distance to the centroid show less individual variation. Statistical differences in these distances were tested using permutest (with 10,000 permutations) in the package vegan. We investigated the differential abundances of bacterial genera that increased significantly under different diets (in the non-rarefied data set) using the DESeq2 package [57]. Differential abundances of bacterial genera were investigated separately for each diet group with their appropriate initial and reversed bacterial communities. To assure that the sequencing depth of different samples did not impact the final outcome of our study, we rarefied the original data set (Additional file 2: Table S2) using the sample with the lowest number of sequences using the vegan [88] and phyloseq [90] packages (Additional file 3: Table S3). Then we performed similar alpha and beta diversity analysis to compare the results of original data set with the rarefied data set.

## Supplementary Information

**Additional file 1 **: **Table S1**. Full ASV table of bacterial sequences with six taxonomic levels. *P. major* individual codes (Table 2) are shown in columns. Codes that end with 1 represent the initial microbial communities, 2–4 represent diet manipulation period, 5 represent the microbial communities at the end of the diet manipulation period, 6–8 represent the diet reversal period and 9 represent the end of the diet reversal period. Individual codes start with “M” were exposed to the mixed diet, “S” was exposed to the seed, while “W” were exposed to mealworm diet during the diet manipulation period. GenBank accession numbers of each sample is provided above the sample code.

**Additional file 2 **: **Table S2**. Subset of the full ASV table only including microbial communities from week 1 (initial microbiomes), week 5 (microbiomes after the diet manipulation), and week 9 (reversed microbiomes). GenBank accession numbers of each sample is provided above the sample code.

**Additional file 3 **: **Table S3**. Rarefied ASV table (only the samples from week 1, week 5 and week 9) with 1045 sequences per sample. Colum names are same as in the Table S2.

**Additional file 4 **: **Table S4**. Dunn’s post-hoc test results for pairwise comparisons between ASV richness and Shannon’s diversity index in the original data (Table S2). Significantly different groups are indicated with asterisks. **Table S5**. Dunn’s post-hoc test results for pairwise comparisons between ASV richness and Shannon’s diversity index in the rarefied data set (Table S3). Significantly different groups are indicated with asterisks.

**Additional file 5 **: **Figure S1**. Five-way Venn diagram to illustrate the shared ASVs in microbiomes under each diet groups. Total number of ASVs found in each group is given within parenthesis under the group name. Pair-wise shared numbers of ASVs are shown below the Venn diagram. Venn diagram was built in http://www.interactivenn.net/ (accessed April 2020).

**Additional file 6 **: **Figure S2**. Genus level trees of seven major bacterial genera with multiple ASVs found in initial, after the diet manipulation and after the diet reversal gut microbiomes. Number of circles in tips represent the number of individuals that each ASV was found and the colour represent the treatment group (initial, mixed, seed, mealworm or reversed).

**Additional file 7 **: **Figure S3**. Alfa and beta diversities of rarefied ASV table (Table S3). **a.** Mean ASV richness and **b.** mean Shannon diversity index of gut microbial communities under initial diet (1st week), after the diet manipulation experiment (5th week) and after the diet reversal (9th week). Results of the Dunn’s post-hoc tests are shown above the box plots (letter differences indicate significant differences between groups). **c.** Non-Metric Multidimensional Scaling (NMDS) plot of rarefied bacterial communities for initial, mixed, mealworm, seed, and reversed diets (ellipses indicate 95% CI; stress = 0.181). Adjusted *p* values and R^2^ values of pair-wise comparisons of adonis analysis (with 10,000 permutations) are given within the figure.

**Additional file 8 **: **Figure S4**. Comparison of individual variation in gut microbiomes of different diet treatments using the average distance of microbial communities to the centroid of the group. Smaller average distances represent groups with low individual variation while longer average distances indicate groups with high individual variations. Significant values of permutation based pairwise comparisons are shown below the boxplot.

**Additional file 9 **: **Figure S5**. Non-Metric Multidimensional Scaling (NMDS) plots of changes in gut bacterial communities during the diet manipulation period on the three diet treatments (a, c, e) and the microbial community changes occur during the 4 weeks of diet reversal period (b, d, f).

**Additional file 10 **: **Table S6**. Results of DeSeq2 analysis on differentially abundant bacterial genera between experimental groups (6.1: Initial diet vs. Mixed diet, 6.2: Mixed diet vs. Reversed diet, 6.3: Initial diet vs. Seed diet, 6.4: Seed diet vs. Reversed diet, 6.5: Initial diet vs. Mealworm diet, and 6.6: Mealworm vs. Reversed diet).

## Data Availability

The data generated and analysed are available from the GenBank SRA database (PRJNA548757).

## Abbreviation

ASV: Amplicon sequence variant
