# Supplementary material for: Flexibility and resilience of great tit (Parus major) gut microbiomes to changing diets
Source: Anim Microbiome. 2021 Feb 18;3:20. doi: 10.1186/s42523-021-00076-6 (PMC7893775; doi:10.1186/s42523-021-00076-6)
Supplement: Supplementary file 4 — Additional file 4 : Table S4. Dunn’s post-hoc test results for pairwise comparisons between ASV richness and Shannon’s diversity index in the original data (Table S2). Significantly different groups are indicated with asterisks. Table S5. Dunn’s post-hoc test results for pairwise comparisons between ASV richness and Shannon’s diversity index in the rarefied data set (Table S3). Significantly different groups are indicated with asterisks. [file 42523_2021_76_MOESM4_ESM.docx]

**Supplementary figures and tables: Additional file 4**

## Flexibility and resilience of Great tit (*Parus major*) gut microbiomes to changing diets

## Kasun H. Bodawatta^1^, Inga Freiberga^2^, Katerina Puzejova^2,3^, Katerina Sam^2,3^, Michael Poulsen^4^, and Knud A. Jønsson^1^

^1^Natural History Museum of Denmark, University of Copenhagen, Copenhagen, Denmark (KHB: [bodawatta@snm.ku.dk](mailto:bodawatta@snm.ku.dk), KAJ: [kajonsson@snm.dk](mailto:kajonsson@snm.dk))

^2^Biology Centre of Czech Academy of Sciences, Institute of Entomology, Ceske Budejovice, Czech Republic (IF: [freiberga.inga@gmail.com](mailto:freiberga.inga@gmail.com), KP: [katypuje@seznam.cz](mailto:Katypuje@seznam.cz), KS: [katerina.sam.cz@gmail.com](mailto:katerina.sam.cz@gmail.com))

^3^Faculty of Science, University of South Bohemia, Ceske Budejovice, Czech Republic

^4^Section for Ecology and Evolution, Department of Biology, University of Copenhagen, Copenhagen, Denmark (MP: [mpoulsen@bio.ku.dk](mailto:mpoulsen@bio.ku.dk))

Corresponding author: Kasun H. Bodawatta, Natural History Museum of Denmark, University of Copenhagen, Copenhagen, Denmark. E-mail: [bodawatta@snm.ku.dk](mailto:bodawatta@snm.ku.dk), Phone: +45 91 72 49 96.

**Supplementary tables 4 and 5**

Table S4. Dunn’s post-hoc test results for pairwise comparisons between ASV richness and Shannon’s diversity index in the original data (Table S2). Significantly different groups are indicated with asterisks.

|  | **ASV richness** | | **Shannon’s diversity index** | |
| --- | --- | --- | --- | --- |
| **Comparison** | **Test statistic (Z)** | **Adjusted p value** | **Test statistic (Z)** | **Adjusted p value** |
| Initial vs. mixed diet | 2.959 | 0.0154* | 3.819 | 0.0013* |
| Initial vs. mealworm diet | 1.803 | 0.1781 | 3.365 | 0.0038* |
| Initial vs. seed diet | 2.437 | 0.0496* | 2.304 | 0.0531 |
| Mixed vs. mealworm diet | -0.7978 | 0.6071 | -0.1641 | 0.8697 |
| Mixed vs. seed diet | -0.2686 | 0.7882 | -1.0499 | 0.3672 |
| Mealworm vs. seed diet | -0.5066 | 0.6805 | 0.8482 | 0.4404 |
| Reversed vs. mixed diet | 0.6768 | 0.6231 | -2.459 | 0.0465* |
| Reversed vs. mealworm diet | -1.543 | 0.2455 | -2.123 | 0.0675 |
| Reversed vs. seed diet | -0.9414 | 0.5775 | -1.115 | 0.3783 |
| Initial vs. reversed diet | 4.651 | <0.001* | 1.533 | 0.2089 |

Table S5. Dunn’s post-hoc test results for pairwise comparisons between ASV richness and Shannon’s diversity index in the rarefied data set (Table S3). Significantly different groups are indicated with asterisks.

|  | **ASV richness** | | **Shannon’s diversity index** | |
| --- | --- | --- | --- | --- |
| **Comparison** | **Test statistic (Z)** | **Adjusted p value** | **Test statistic (Z)** | **Adjusted p value** |
| Initial vs. mixed diet | 3.987 | 0.0007* | 3.749 | 0.0018* |
| Initial vs. mealworm diet | 3.549 | 0.0013* | 3.378 | 0.0036* |
| Initial vs. seed diet | 2.341 | 0.0482* | 2.347 | 0.0474* |
| Mixed vs. mealworm diet | -0.1415 | 0.8874 | -0.0984 | 0.9216 |
| Mixed vs. seed diet | -1.151 | 0.4996 | -0.9596 | 0.4215 |
| Mealworm vs. seed diet | 0.9663 | 0.4769 | 0.8245 | 0.4551 |
| Reversed vs. mixed diet | -0.9937 | 0.5339 | -2.446 | 0.0481* |
| Reversed vs. mealworm diet | -0.7724 | 0.5498 | -2.186 | 0.0576 |
| Reversed vs. seed diet | 0.3757 | 0.7857 | -1.206 | 0.3254 |
| Initial vs. reversed diet | 3.711 | 0.0011* | 1.461 | 0.2403 |
