# Supplementary material for: Flexibility and resilience of great tit (Parus major) gut microbiomes to changing diets
Source: Anim Microbiome. 2021 Feb 18;3:20. doi: 10.1186/s42523-021-00076-6 (PMC7893775; doi:10.1186/s42523-021-00076-6)
Supplement: Supplementary file 5 — Additional file 5 : Figure S1. Five-way Venn diagram to illustrate the shared ASVs in microbiomes under each diet groups. Total number of ASVs found in each group is given within parenthesis under the group name. Pair-wise shared numbers of ASVs are shown below the Venn diagram. Venn diagram was built in http://www.interactivenn.net/ (accessed April 2020). [file 42523_2021_76_MOESM5_ESM.pdf]

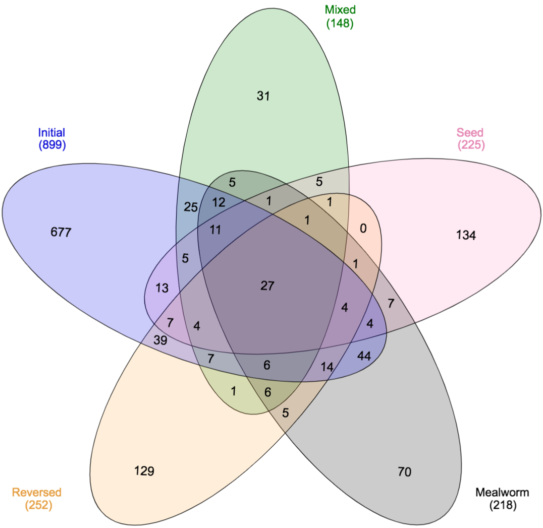

Pairwise shared number of ASVs between different groups

	Reversed	Initial	Mixed	Seed
Initial	108			
Mixed	53	97		
Seed	45	75	58	
Mealworm	64	122	71	58
