# Supplementary figures and images for: Flexibility and resilience of great tit (Parus major) gut microbiomes to changing diets
Source: Anim Microbiome. 2021 Feb 18;3:20. doi: 10.1186/s42523-021-00076-6 (PMC7893775; doi:10.1186/s42523-021-00076-6)

*Enterococcus*

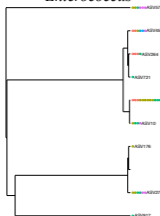

*Lactobacillus*

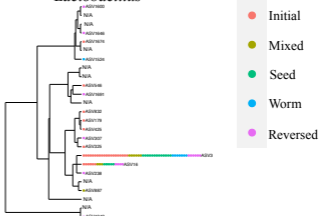*Weissella*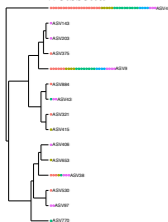

*Lactococcus*

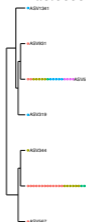

*Pseudomonas*

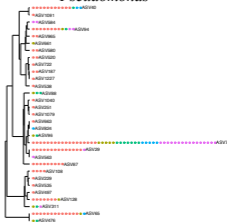*Massilla*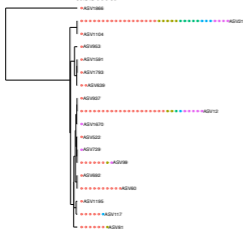

*Acinetobacter*

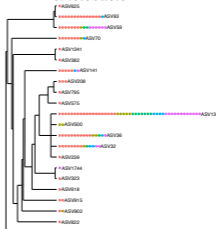

Supplement: Supplementary file 6 — Additional file 6 : Figure S2. Genus level trees of seven major bacterial genera with multiple ASVs found in initial, after the diet manipulation and after the diet reversal gut microbiomes. Number of circles in tips represent the number of individuals that each ASV was found and the colour represent the treatment group (initial, mixed, seed, mealworm or reversed). [file 42523_2021_76_MOESM6_ESM.pdf]

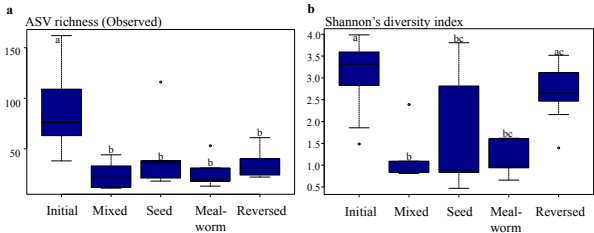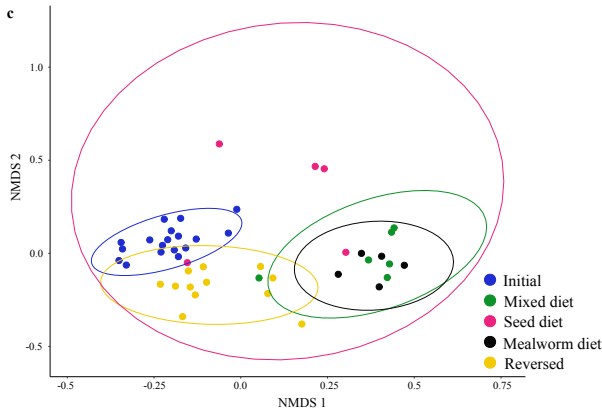

Community level  
differences  
(pairwiseAdonis with  
10,000 permutations)

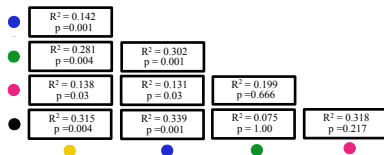

Supplement: Supplementary file 7 — Additional file 7 : Figure S3. Alfa and beta diversities of rarefied ASV table (Table S3). a. Mean ASV richness and b. mean Shannon diversity index of gut microbial communities under initial diet (1st week), after the diet manipulation experiment (5th week) and after the diet reversal (9th week). Results of the Dunn’s post-hoc tests are shown above the box plots (letter differences indicate significant differences between groups). c. Non-Metric Multidimensional Scaling (NMDS) plot of rarefied bacterial communities for initial, mixed, mealworm, seed, and reversed diets (ellipses indicate 95% CI; stress = 0.181). Adjusted p values and R2 values of pair-wise comparisons of adonis analysis (with 10,000 permutations) are given within the figure. [file 42523_2021_76_MOESM7_ESM.pdf]
