# Supplementary material for: Flexibility and resilience of great tit (Parus major) gut microbiomes to changing diets
Source: Anim Microbiome. 2021 Feb 18;3:20. doi: 10.1186/s42523-021-00076-6 (PMC7893775; doi:10.1186/s42523-021-00076-6)
Supplement: Supplementary file 8 — Additional file 8 : Figure S4. Comparison of individual variation in gut microbiomes of different diet treatments using the average distance of microbial communities to the centroid of the group. Smaller average distances represent groups with low individual variation while longer average distances indicate groups with high individual variations. Significant values of permutation based pairwise comparisons are shown below the boxplot. [file 42523_2021_76_MOESM8_ESM.pdf]

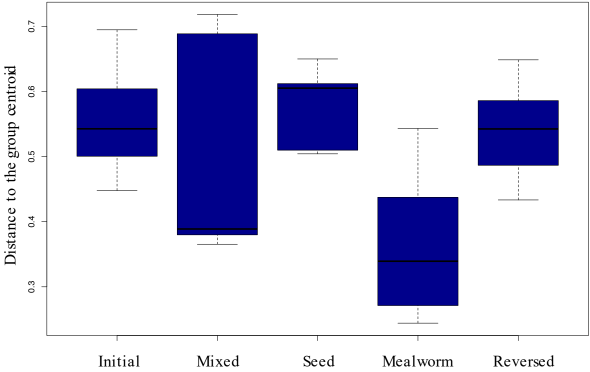

Pairwise differences in distance to the group centroid

	Reversed	Initial	Mixed	Seed
Initial	p = 0.6115			
Mixed	p = 0.3557	p = 0.1732		
Seed	p = 0.3420	p = 0.5222	p =0.2995	
Mealworm	**p =0.0021**	**p =0.0002**	p = 0.2129	**p =0.0082**
