# Supplementary material for: Flexibility and resilience of great tit (Parus major) gut microbiomes to changing diets
Source: Anim Microbiome. 2021 Feb 18;3:20. doi: 10.1186/s42523-021-00076-6 (PMC7893775; doi:10.1186/s42523-021-00076-6)
Supplement: Supplementary file 9 — Additional file 9 : Figure S5. Non-Metric Multidimensional Scaling (NMDS) plots of changes in gut bacterial communities during the diet manipulation period on the three diet treatments (a, c, e) and the microbial community changes occur during the 4 weeks of diet reversal period (b, d, f). [file 42523_2021_76_MOESM9_ESM.pdf]

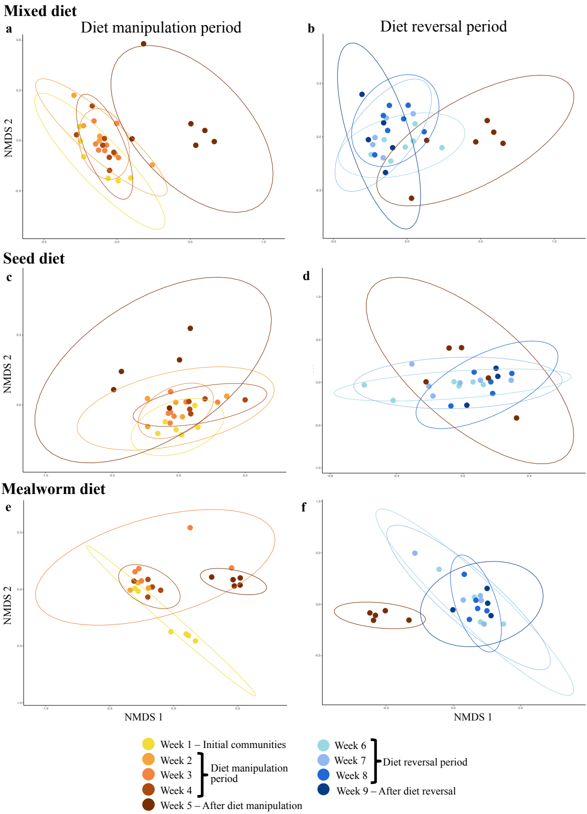

Mixed diet
a
Diet manipulation period
b
Diet reversal period
Seed diet
c
d
Mealworm diet
e
f
NMDS 2
NMDS 1
Week 1 – Initial communities
Week 2
Week 3
Week 4
Diet manipulation period
Week 5 – After diet manipulation
Week 6
Week 7
Week 8
Diet reversal period
Week 9 – After diet reversal
